# Design of High-Performance Electrospun Membranes for Protective Clothing Applications

**DOI:** 10.3390/membranes14110244

**Published:** 2024-11-20

**Authors:** Anca Filimon, Diana Serbezeanu, Daniela Rusu, Alexandra Bargan, Lavinia Lupa

**Affiliations:** 1 “Petru Poni” Institute of Macromolecular Chemistry, Grigore Ghica Alley 41A, 700487 Iasi, Romania; afilimon@icmpp.ro (A.F.); rusu.daniela@icmpp.ro (D.R.); anistor@icmpp.ro (A.B.); 2 Faculty of Industrial Chemistry and Environmental Engineering, Politehnica University Timisoara, 6 Vasile Parvan Blv, 300223 Timisoara, Romania; lavinia.lupa@upt.ro

**Keywords:** polyimides, polyether ether ketone, electrospun membranes, breathability, impermeability, protective clothing

## Abstract

The integration of nanomaterials into the textile industry has significantly advanced the development of high-performance fabrics, offering enhanced properties such as UV blocking, fire resistance, breathability, hydrophobicity, antimicrobial activity, and dust rejection. In this context, our research explores the development and characterization of electrospun membranes composed of polyether ether ketone (PEEK) and various polyimides (PIs (1–6)), focusing on their application in protective clothing. The combination of phosphorus-containing polyimides and PEEK, along with the electrospinning process, enhances the distinctive properties of both PEEK and polyimides, leading to composite membranes that stand out according to key parameters essential for maintaining physiological balance. The structural and morphological characteristics of these membranes have been evaluated using Fourier transform infrared spectroscopy (FTIR) to identify the functional groups and scanning electron microscopy (SEM) to examine their morphology. These analyses provide critical insights into these materials’ properties, which influence key performance parameters such as moisture management, breathability, and barrier functions. The membranes’ breathability and impermeability were assessed through the water vapor transmission rate (WVTR), contact angle measurements, water and air permeability, and flame resistance tests. The results obtained indicate that PEEK/polyimide composite membranes meet the complex requirements of modern protective textiles, ensuring both safety and comfort for users through their optimized structural properties and enhanced functional capabilities.

## 1. Introduction

The skin functions as an exceptional natural defense system, acting as a shield from environmental challenges, such as extreme temperatures, chemicals, and physical stressors, maintaining the body’s resilience [1]. However, due to its sensitivity, additional protective measures are often required, and in this regard, clothing plays a key role, offering not just physical protection but also thermal regulation and sensory comfort. To ensure this optimal comfort, it is crucial to establish a microclimate between the skin and the fabric in order to prevent skin irritations, allergic reactions, and direct exposure to harmful agents [2]. This microclimate’s balance can be influenced by external factors such as, for example, temperature and humidity, which can disrupt the comfort of the wearer if not properly managed. Effective regulation of perspiration is essential, as the evaporation of sweat requires heat absorption to stabilize body temperature [3]. Clothing facilitates this process by allowing moisture and heat to pass through, thus maintaining a stable temperature and humidity within the air layer next to the skin.

Natural fibers, like cotton and wool, are known to be particularly effective in this role due to their high capacity to create natural air flow and ventilation [4]. In the early 1960s, the rising interest in more affordable and recyclable synthetic fibers like polyester led to a decline in the demand for natural fibers, significantly reducing the market share of the natural fiber industry [5]. This shift has driven the market towards more cost-effective synthetic alternatives. However, the limitations of traditional synthetic fibers—specifically their inability to mimic the breathability and air-trapping qualities of natural fibers—prompted the textile industry to seek more advanced materials that could enhance the performance of fabric in various applications, including clothing, medical uses, and environmental solutions [6,7]. This push for innovation has increasingly involved the use of nanotechnology, allowing for the creation of high-performance fabrics with miniaturized structures that can adapt to changing conditions [8]. In this context, polymers present significant opportunities to create adaptive materials capable of responding to microclimate needs through mechanisms that are still unexplored [9], and designed membranes are examples of this innovation, functioning as heat exchangers, water condensers, and protective barriers against contaminants. These advancements in material science and nanotechnology hold great potential for improving our quality of life, health, and overall lifestyle by offering enhanced comfort and functionality in various environments.

As a result of these considerations, the quest of the development of advanced materials for protective clothing that offer both superior performance and functional versatility has led to significant advancements in material science [10,11,12,13]. Among the promising materials for this purpose, polyimides (PIs) and poly(ether-ether-ketones) (PEEKs) have emerged as outstanding candidates due to their thermal stability, mechanical strength, and chemical resistance [12,14]. Polyimides that contain phosphorus are particularly noteworthy due to their flame-retardant properties, making them invaluable in high-performance applications [15,16]. When combined with PEEK, a polymer renowned for its durability and high-temperature resistance [12,17,18,19,20,21], the resultant composite materials promise enhanced functionality as a result of the acquired properties, positioning them as key materials in the design of high-performance protective clothing that meets the stringent requirements of modern safety standards [10,12,21,22]. Thus, the combination of PEEK’s inherent robustness with the flexibility and thermal stability of polyimides results in membranes that can endure harsh environmental conditions while maintaining the desired balance between breathability and waterproofness. Studies have shown [12,19,22,23,24,25] that by optimizing the composition and structure of these composite membranes, it is possible to achieve the desired transport properties without compromising their protective capabilities. This makes them particularly suitable for use in environments where both protection and comfort are critical, such as in firefighting gear, chemical protective suits, and outdoor sportswear.

In addition, recent advancements in membrane technology, particularly through electrospinning, a technique that creates fine fibers from polymer solutions, has become an essential method for fabricating high-performance materials/membranes with unique characteristics suitable for demanding environments [26,27,28]. The versatility of electrospinning allows for precise control over the diameter, porosity, and thickness of fibers, all of which directly impact the permeability and breathability of the final membrane [29,30]. In other words, this technique allows for precise tracking of a material’s morphology and properties, which is advantageous for controlling the transport of air and water vapor—essential properties for developing breathable protective clothing that allows the body to maintain its physiological balance, permits the removal of moisture vapor while preventing water penetration, and thus keeps the wearer/user dry and comfortable even in demanding conditions [31,32,33,34].

The ability to modulate the transport properties of water vapor and air is crucial in achieving the desired balance between breathability and waterproofness in protective membranes [35,36]. For this reason, the combination of phosphorus-containing polyimides and PEEK through electrospinning has been explored to capitalize on the strengths of each of these two polymers, aiming to develop electrospun membranes that are distinguished by critical parameters such as their mechanical strength, thermal management, and flame resistance. This procedure was chosen knowing that blending PEEK with polyimides or polyetherimides has been reported to be an effective way to combine the properties of both polymers, resulting in materials with desirable characteristics, such as improved chemical resistance, thermal stability, and mechanical properties, for high-performance applications [10,12,14]. Enhancing the water and air transport properties of electrospun PEEK/polyimide membranes represents the focal point in our research in order to create high-performance protective materials. By carefully adjusting the structure and morphology of electrospun PEEK/polyimide membranes, we sought to tune these transport properties, ensuring that the membranes provide an optimal barrier to external moisture while allowing for efficient moisture release from the body. This modulation not only enhances the comfort of protective clothing but also plays a critical role in maintaining the physiological well-being of the wearer by preventing overheating and moisture accumulation.

## 2. Materials and Methods

### 2.1. Materials

The materials used for synthesis were purchased from Sigma-Aldrich Chemie GmbH: 4,4′-Oxydiphthalic anhydride (97%, Mw = 310.21 g/mol), 2,2-bis [4-(3,4-dicarboxyphenoxy) phenyl]propane dianhydride (98%, Mw = 410.51 g/mol), 4,4′-(hexafluoroisopropylidene) diphthalic anhydride (99%, Mw = 444.24 g/mol), 4,4′-diaminobenzophenone (97%, Mw = 212.25 g/mol), phenolphthalein, 4,4′-difluorobenzophenone (purity: 98.5%), potassium carbonate (ACS reagent, 99%), and *N*-methyl-2-pyrrolidone (NMP) (HPLC, 99.9% purity). 9,10-Dihydrooxa-10-phosphaphenanthrene-10-oxide (DOPO) (97%, Mw = 216.18 g/mol), obtained from TCI (Japan), was dehydrated under a vacuum at 120 °C for 5 h before use. Synthesis of DOPO-diamine (1a) was carried out following the established procedures using 4,4′-diaminobenzophenone and 9,10-dihydro-oxa-10-phosphaphenanthrene-10-oxide as the starting materials [37]. The reaction yield was 75%. ^1^H NMR (DMSO-d_6_, ppm): δ = 4.9 (s, 4H, NH_2_), 5.9 (d, 4H, CH_ar-NH_2_), 8.5–6.9 (m, 20H, aromatic protons). ^31^P NMR (DMSO-d_6_, ppm): δ = 31.10 and 29.41. Diamine 1b, bis(3-aminophenyl)-methyl-phosphine oxide (BAPMPO), was obtained at a yield of 75%. ^1^H NMR (DMSO-d_6_, ppm): δ = 1.8 (d, 3H, CH_3_), 5.38 (s, 4H, NH_2_), 6.69–6.67 (d, 2H, CH_ar-NH_2_), 6.9–6.7 (m, 2H, aromatic protons), 6.91–6.88 (d, 2H, aromatic protons), 7.14–7.11 (m, 2H, aromatic protons). ^31^P NMR (DMSO-d_6_, ppm): δ = 28.66–28.43 [38]. All of the other solvents necessary for the synthesis, purification, and precipitation of the polymers were used as received.

### 2.2. Synthesis of the Polymers

**Scheme 1:**
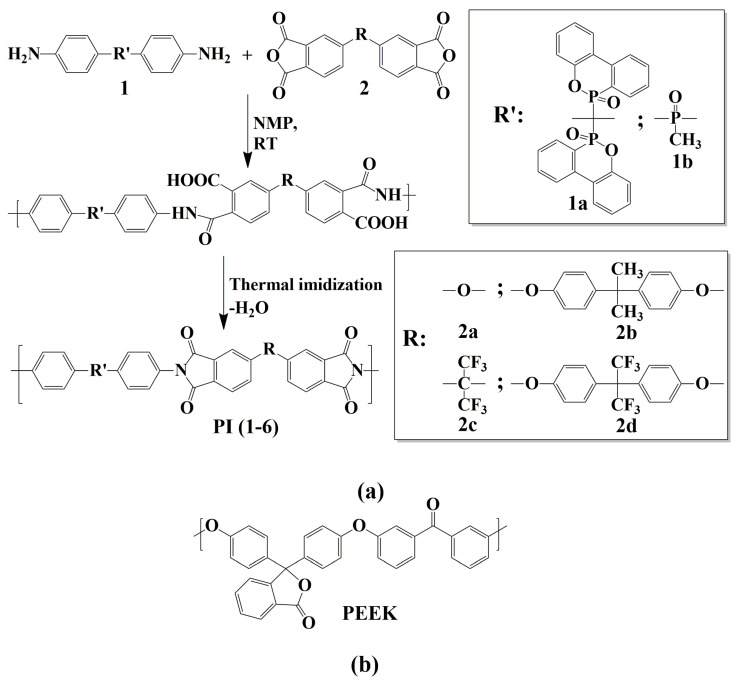
(**a**) Synthesis of PIs (1–6) and (**b**) chemical structure of PEEK.

The aromatic polyimides PIs (1–6) were synthesized through a two-step polycondensation reaction, involving the diamines labeled 1a and 1b with four commercial aromatic dianhydrides—phthalic anhydride, 2a; 2,2-Bis(4-(4-aminophenoxy)phenyl)propane, 2b; 4,4′-(hexafluoroisopropylidene)diphthalic anhydride, 2c; or 2,2-Bis [4-(4-aminophenoxy)phenyl]hexafluoropropane, 2d—using NMP as a solvent. The first step involved the opening of the anhydride ring at a low temperature, leading to the formation of a pre-polymer of the polyamide type with carboxylic groups. The second step consisted of the cyclodehydration of the polyamide acid obtained in the previous step at elevated temperatures, forming the corresponding polyimide structure. The water produced was removed from the system under a gentle nitrogen stream, shifting the equilibrium toward the formation of the macromolecular imide compound (Scheme 1a). The synthesis method of polyimides PI-1–PI-3 is detailed in the article [15]. PEEK was synthesized using a mixture of phenolphthalein (3.498 g, 0.011 mol), 4,4′-difluorobenzophenone (2.398 g, 0.011 mol), potassium carbonate (0.022 mol), toluene (10 mL), and NMP (27 mL). The mixture was stirred and heated to 140–150 °C for 5 h to remove water by azeotropic distillation. Toluene was then distilled off, and the reaction continued at 180 °C for 8 h. After cooling, the obtained product was diluted with NMP and precipitated in HCl, followed by filtration, washing, and drying. The structure of the PEEK polymer is shown in Scheme 1b. The polymer had an Mn of 51,000 g/mol (PDI = 1.89). The synthesis method was adapted from a previously published article [12].

### 2.3. Electrospinning Solutions

For the preparation of the PEEK/PI (1–6) nanofibrous membranes, 0.1 g of each polyimide (PIs (1–6)) was added to 2 mL of a 15% *w*/*v* PEEK solution in NMP. The mixtures were stirred thoroughly to ensure complete dissolution and homogeneity. Once they were fully mixed, the resulting solutions were subjected to electrospinning under controlled conditions to fabricate nanofibrous membranes. During electrospinning, the polymer solution was placed in a syringe and expelled through a needle under a high-voltage electric field, forming fine polymer fibers that were collected onto a grounded collector. The process resulted in uniform nanofibrous membranes composed of PEEK/PI (1–6) blends.

### 2.4. Electrospinning Process

Using a Fluidnatek^®^ LE-50 laboratory system from Bioinicia S.L. (Valencia, Spain), the electrospun fibers were obtained from the homogeneous PEEK/PI (1–6) solutions in NMP. The process was carried out under the following conditions: an applied voltage of 21.7–24.6 kV, a needle-to-collector distance of 16–20 cm, and a solution flow rate of approximately 20 µL/min with a 27 G stainless steel needle. The electrospun PEEK/PI (1–6) fibers were deposited onto a static copper grid covered with a backing foil sheet, which served as the collector. The fiber collection took place over a duration of about 2 h, with the ambient temperature maintained between 23 and 25 °C.

### 2.5. Rheological Measurements

The dynamic rheological behavior of the PI (1–6) solutions in NMP was analyzed using a Bohlin Instrument CS-50 rheometer (Malvern Instruments, Worcestershire, UK). The measurements utilized a cone–plate geometry with a 4° cone angle and a diameter of 40 mm. Shear viscosity was measured over a shear rate range of 0.07–50 s^−1^ at 25 °C, with all the rheological data recorded to within ±5% accuracy for each measurement.

### 2.6. Fourier Transform Infrared Spectroscopy with Analysis of the Attenuated Total Reflectance

The infrared spectra of the electrospun PEEK/PI (1–6) membranes were obtained using a Bruker LU-MOS FTIR Microscope (Bruker Optik GmbH, Ettlingen, Germany), equipped with an attenuated total reflection (ATR) module featuring a diamond crystal, and processed using OPUS 8 software (Version 8, Ettlingen, Germany). A single reflection at a 45° angle was employed for spectral processing. The scanning range for each sample was set between 600 and 4000 cm^−1^. All spectra were recorded at a temperature of 25 °C.

### 2.7. ^1^H NMR Spectroscopy

The ^1^H NMR spectra of the monomers and polymers investigated in this study were recorded at 25 °C in DMSO-d_6_ using a Bruker Avance DRX 400 spectrometer (Rheinstetten, Germany).

### 2.8. Scanning Electron Microscopy

The morphology of the electrospun PEEK/PI (1–6) membranes was analyzed using scanning electron microscopy (SEM) with a Quanta 200 ESEM device operating at 5 kV and 10 kV, with magnifications ranging from 380 to 3600. Prior to imaging, the samples were coated with a thin layer of gold using an EK 3135 EMITECH sputter coater.

### 2.9. Thickness, Apparent Density, and Porosity

The thickness of the electrospun PEEK/PI (1–6) membranes was measured using a digital micrometer. The apparent density and porosity of the new electrospun PEEK/PI (1–6) membranes were calculated using Equations (1) and (2):

(1) $$\rho_{app}=m/d\;A$$

(2) $$\varepsilon =((1-\rho_{app})/\rho )\cdot 100\;$$

where the parameters involved were *ρ_app_*, the apparent density (g/cm^3^); *m*, the mass of the membrane (g); *d*, the thickness of the membrane (cm); *A*, the area of the membrane (cm^2^); *ε*, porosity (%); and *ρ*, the density of the sample (g/cm^3^).

### 2.10. Water Vapor Transmission Rate

The water vapor transmission through the electrospun PEEK/PI (1–6) membranes was measured using a water vapor transmission tester (model W3/031, Jinan Languang Electromechanical Technology Co., Ltd., Jinan, China).

### 2.11. Contact Angle Measurements

The sessile-drop method was employed to measure the contact angles using a CAM-101 system (KSV Instruments, Helsinki, Finland) [39]. For a statistical evaluation, different regions of the electrospun PEEK/PI (1–6) membranes’ surfaces were selected and the contact angle between the membrane and water measured there. Three measurements were taken, and the average contact angle was calculated with an error of ±1%.

### 2.12. Water Permeability

Water permeability was assessed using a pressurized static method. In this procedure, a constant water pressure of 20 kPa was applied to one side of the electrospun PEEK/PI (1–6) membranes. The time required for water to traverse from one side of the membrane to the other was meticulously measured. Consequently, membranes with a diameter of 1 cm and a height of 60 µm were positioned within a filtration apparatus and immersed in 50 mL of distilled water. A pressure of 20 kPa or greater was applied, and the time necessary for water to permeate the membrane was recorded. The permeation rate was subsequently calculated using Equation (3) [40]:

(3) J=V/(A · t)

where *J* is denoted as the permeation rate, *V* is the volume flowing through the membrane, *A* the area of the membrane, and *t* the time.

### 2.13. Air Permeability

Air permeability, which indicates a material’s ability to allow air to pass through it, was determined according to STAS 5902-1970 or ASTM D737 using an ATL-2 setup (Metrimpex, Hungary).

### 2.14. The UL-94 Test

The vertical burning test (UL-94) was conducted on test specimens (130 × 13 × 3 mm^3^), which were suspended vertically above a cotton patch to detect any burning droplets. The classifications were assigned based on the American National Standard UL 94-2006.

## 3. Results and Discussion

### 3.1. Structural Characterization and Thermal Stability of Polyimides

The formation of polyimides PIs (1–6) was confirmed using FTIR spectroscopy [15]. The disappearance of the amide bands at 1670 cm^−1^ indicates the conversion of ortho-carboxy-amide groups into imide rings. In the FTIR spectra of PIs (1–6), the appearance of strong bands at 1780 cm^−1^ and 1720 cm^−1^ was observed, which were attributed to asymmetric and symmetric vibrations of the carbonyl groups in the imide ring. Absorption bands at 1370 cm^−1^, 740 cm^−1^, and 1240 cm^−1^ were assigned to the C-N vibrations of the imide ring, imide ring deformation vibrations, and the aromatic ether bond (Ar-O-Ar), respectively. Polymers PI-3 and PI-4 exhibited characteristic absorption bands at 1180 cm^−1^ and 1210 cm^−1^ corresponding to the 6F groups [15].

Polymers PIs (1–4) lose 5% of their weight within the temperature range of 179–394 °C due to the decomposition of their side groups (Table 1). Their lower thermal stability, compared to polyimides without bulky DOPO groups, is attributed to the weak P–O bond in the phosphorus-containing heterocycle. However, the introduction of bulky DOPO groups enhances the stability of the carbonaceous residue. Polymers PI-5 and PI-6 exhibit thermal stability, beginning to decompose within the temperature range of 447–460 °C.

The temperature at which they experience a 10% weight loss is approximately 485 °C, indicating their resistance to thermal degradation. At 750 °C, the carbonaceous residue, a measure of the material’s char yield and a key indicator of thermal stability, was 47.29% for PI-5 and 61.36% for PI-6. These values suggest that PI-6 has superior thermal stability to PI-5. Both polymers also show high values of the Limiting Oxygen Index (LOI), a crucial indicator of flame retardancy (Table 1). The LOI values for the materials studied were equal to 34.5 and 42, with PI-6 achieving the highest LOI value, signifying its excellent fire-resistant properties. The chemical structures of these polyimides influence their thermal and flame-retardant properties. The presence of the phosphine oxide group in both polymers contributes to their high thermal stability and flame-retardant characteristics, with PI-6’s structure imparting an enhanced performance in these areas due to the additional rigidity and cross-linking potential provided by the 2,2-Bis(4-(4-aminophenoxy)phenyl)propane unit.

### 3.2. Role of the Solution and Environmental Parameters in the Manufacturing Process of PEEK/PI Membranes

The phosphorus-containing polymers were subjected to the electrospinning process. Based on the studies conducted, the optimal conditions for preparing membranes with “ideal” properties using the electrospinning process were determined. The optimal conditions for membrane fabrication, along with the viscosity and average fiber diameters for the samples that successfully produced fibers, are summarized in Table 2. The average fiber diameters were calculated using ImageJ software (Version 1.46, 14 December 2018) and are also presented in Table 2, providing a clear comparison of the fiber morphology across different samples. These values reflect the precision and consistency of the electrospinning process under the given parameters, with the resulting fiber diameters offering insights into the quality and structure of the nanofibrous membranes.

**Figure 1:**
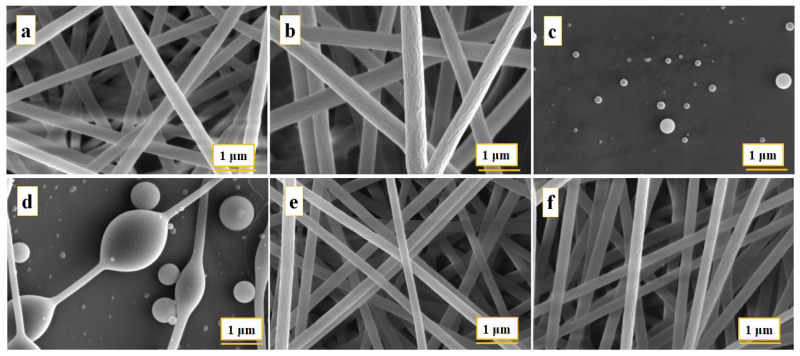
SEM images of the electrospun fibers, (**a**) PI-1, (**b**) PI-2, (**c**) PI-3, (**d**) PI-4, (**e**) PI-5, and (**f**) PI-6, recorded with a magnification of 20,000× in a 1 μm scan area.

Figure 1 displays SEM images of electrospun fibers from different polyimide (PI) samples—PI-1 (a), PI-2 (b), PI-3 (c), PI-4 (d), PI-5 (e), and PI-6 (f)—where the various fiber morphologies were obtained based on the polyimide type. Thus, the PI-1 and PI-2 samples exhibit uniform, smooth fibers with consistent diameters, indicating stable electrospinning conditions and successful fiber formation (Figure 1a,b). Instead, PI-3 and PI-4 (Figure 1c,d) show the presence of beads or droplets, which indicates problems during electrospinning, such as an improper viscosity, surface tension, or flow rate. These defects may arise from solution instability or insufficient polymer chain entanglement.

On the other hand, PI-5 and PI-6 display well-formed fibers with relatively consistent diameters and minimal defects, suggesting optimized electrospinning parameters for these polyimide compositions (Figure 1e,f). The differences in fiber morphology in these images highlight the importance of tuning the electrospinning parameters, such as the solution viscosity, concentration, and applied voltage, to achieve high-quality nanofibrous membranes (Table 2).

Obtaining electrospun membranes with the desired structure and specific properties that meet the complex requirements of protective fabrics implies the appropriate choice of polymeric material. Thus, the use of PEEK provides a strategic approach to modulating the transport properties of water vapor and air, enabling the creation of breathable protective phosphorus-containing-polyimide-based-membranes by blending it with PIs (1–6). This balance is crucial to ensure physiological comfort through effective moisture management while maintaining a high-performance capacity for the filtration of aerosol particles, chemical vapors, and other potentially harmful species, thereby supporting both physiological balance and environmental protection. For this, PEEK was dissolved in NMP, and the solution obtained was then mixed with the six polyimides studied (PIs (1–6)). The electrospinning conditions and the average fiber diameters for the phosphorus-containing polyimide membranes, which are self-standing, are detailed in Table 3.

**Figure 2:**
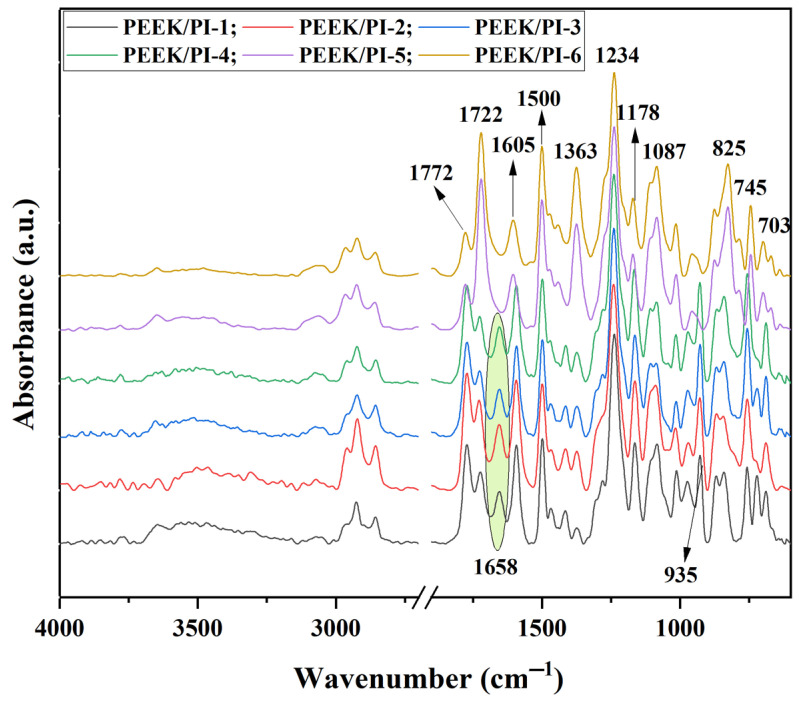
FTIR spectrum of the electrospun PEEK/PI (1–6) membranes.

The FTIR spectra of the electrospun PEEK/PI (1–6) membranes investigated, shown in Figure 2, reveal characteristic absorption bands of both electrospun PEEK fibers and their mixtures. Thus, for the neat PEEK fibers, notable peaks include those at 1772 cm^−1^ and 1646 cm^−1^ (C=O stretching), 1605 cm^−1^ (skeletal in-plane phenyl ring vibrations), 1495 cm^−1^ (aromatic rotational vibrations), 1234–1178 cm^−1^ (diphenyl ether C–O–C stretching), 928 cm^−1^ (aromatic out-of-plane bending), and 825 and 745 cm^−1^ (C–H out-of-plane bending) [12]. Upon the incorporation of the phosphorus-containing polyimide (PI), distinct new bands emerge at 1722 cm^−1^, 1363 cm^−1^, and 935 cm^−1^, signaling the successful integration of PIs (1–4) into the composite structure. These bands correspond to the characteristic vibrations of the polyimides and confirm their presence within the PEEK matrix.

Additionally, the peaks at 1470 cm^−1^ (P–Ph), 1234 cm^−1^ (P=O), and 1178 cm^−1^ and 935 cm^−1^ (P–O–Ph) are indicative of the incorporation of the DOPO groups, a hallmark of phosphorus-modified polyimides. The absorption band observed at 1658 cm^−1^, unique to the electrospun PEEK/PI (1–4) membranes, likely results from specific interactions between the PEEK matrix and the phosphorus-containing polyimide. This could be attributed to imide group vibrations or intermolecular interactions induced by the presence of DOPO, which modify the spectral signature of the composite. In the cases of PEEK/PI-3 and PEEK/PI-4, the presence of the –6F group (hexafluoroisopropylidene) introduces another characteristic feature into the FTIR spectra. Fluorine-containing groups typically exhibit C–F stretching vibrations in the 1200–1000 cm^−1^ range, with strong absorption bands, particularly between 1100 and 1140 cm^−1^, corresponding to symmetric and asymmetric C–F stretching. Thus, in the spectra of PEEK/PI-3 and PEEK/PI-4, additional peaks or intensified absorption bands in this region reflect the presence of the fluorinated –6F component, providing clear evidence of its incorporation into the composite fibers. These fluorine-related peaks are distinguishable from other functional group vibrations, confirming the successful integration of the hexafluoroisopropylidene structure within the PEEK matrix. In contrast, the PEEK/PI-5 and PEEK/PI-6 fibers, where phosphorus is part of the main polymer chain rather than incorporated through DOPO side groups, exhibit insignificant spectral differences. While the C=O stretching band at 1722 cm^−1^ remains, characteristic DOPO-related peaks, such as the 1470 cm^−1^ (P–Ph) and 1234 cm^−1^ (P=O) bands, are less pronounced or absent. These membranes exhibit slight shifts and modifications in the 1360–950 cm^−1^ region, reflecting phosphorus being more integrated within the polymer backbone. This indicates the subtle influence of phosphorus in the FTIR spectra of the composites compared to the DOPO-containing counterparts, highlighting the differences in how the phosphorus is incorporated and interacts within the PEEK/PI system.

**Figure 3:**
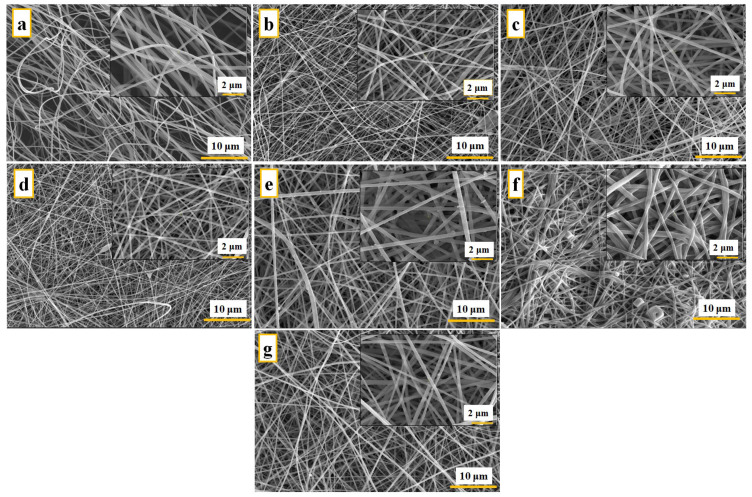
SEM images of the electrospun membranes, (**a**) PEEK, (**b**) PEEK/PI-1, (**c**) PEEK/PI-2, (**d**) PEEK/PI-3, (**e**) PEEK/PI-4, (**f**) PEEK/PI-5, and (**g**) PEEK/PI-6, recorded with a magnification of 2500× in a 10 μm scan area. Small images, obtained with a magnification of 10,000× in a 2 μm scan area, were inserted for better visualization of the nanofiber formation.

Based on these remarks, electrospinning of the PEEK/PI (1–6) solutions was performed, and the SEM images recorded indicate the morphology of the electrospun PEEK and PEEK/PI (1–6) membranes (Figure 3). The PEEK fibers (Figure 3a) appear uniform and continuous, with no major defects visible. The fibers’ diameter is 0.211 ± 0.048 μm, meaning the fibers are fairly uniform with a small variation in diameter. Each image (Figure 3b–g) shows variations in the fiber morphology based on the polyimide used. For instance, the average fiber diameter of PEEK/PI-1 is 0.157 ± 0.032 μm, indicating finer fibers compared to those of PEEK, which may be attributed to the increased chain mobility imparted by the polyimide. The PEEK/PI-2 membranes present a slightly larger diameter of 0.234 ± 0.047 μm, suggesting that the specific properties of PI-2 could contribute to the formation of thicker fibers. Interestingly, PEEK/PI-3 shows the smallest average fiber diameter at 0.135 ± 0.029 μm, which may be due to the enhanced solubility or lower viscosity of the electrospinning solution, allowing for finer fiber formation. Conversely, PEEK/PI-4 and PEEK/PI-5 display larger average fiber diameters of 0.300 ± 0.028 μm and 0.293 ± 0.069 μm, respectively. This increase could be indicative of the polyimide’s molecular structure leading to a higher viscosity, which hinders fiber elongation during electrospinning. Lastly, PEEK/PI-6 yields an average fiber diameter of 0.252 ± 0.062 μm, indicating a balance between the effects of the polyimide structure and the electrospinning parameters. The membranes made using PEEK/PIs (1–6) are white and flexible and have a thickness ranging from 0.02 to 0.08 mm depending on the electrospinning time.

Moreover, the porosity (*ε*) indicates the volume fraction of voids or pores in a membrane and determines the material’s ability to allow air and moisture to pass through. A higher porosity typically correlates with better permeability and breathability. In the case of the electrospun membranes, PEEK/PI-1 (54.7%) has the lowest porosity, while PEEK/PI-3 (87.2%) has the highest (Table 4).

The side-chain nature of the phosphorus in DOPO might make the polymer chains more flexible and compact during electrospinning, resulting in denser fibers with lower porosity. We observed that the bulky -CF_3_ groups present in the electrospun PEEK/PI-3 membrane disrupted chain packing, leading to more porous fibers. This increased porosity is correlated with the smaller diameter of these fibers, as it allows for enhanced chain mobility during the electrospinning process. PEEK/PI-1 and PEEK/PI-2 show a greater porosity compared to PEEK/PI-3 and PEEK/PI-4, which contain both DOPO and -CF_3_ groups. This indicates that the combination of side-chain phosphorus and bulky groups (like -CF_3_) increases the porosity, as seen in PEEK/PI-3 and PEEK/PI-4.

### 3.3. Assessing the Membrane’s Breathability and Permeability: Water Vapor Transport and Wettability

Hydrophobic materials are generally preferred for creating microporous membranes, as they provide an effective barrier to liquid water while allowing water vapor to pass through due to their pore structure. In contrast, hydrophilic polymers are commonly employed in dense laminates to enhance water affinity and support water vapor transmission through adsorption–diffusion mechanisms, contributing to breathability. For applications requiring physiological comfort, breathable membranes must balance moisture transmission with the ability to filter aerosol particles, chemical vapors, or potentially harmful species effectively. A breathable membrane is defined by its capacity to permit water vapor passage while blocking liquid water entry. Typically, “breathable” refers to membranes with a water vapor permeability exceeding 400 g/m^2^·day, as measured by standards such as ASTM E96 [41,42,43]. In this context, assessment of a material’s ability to breathe, in particular that of the electrospun PEEK/PI (1–6) membranes, was carried out according to the transport of water vapor, which is a crucial parameter in evaluating the comfort characteristics of a fabric (sweat transfer). As shown in Table 4, the water vapor transmission rate (WVTR) ranged from 1889.59 ± 110.73 to 2311.20 ± 206.58 (g/m^2^·day), which represented significantly higher values compared to those reported for firefighter clothing materials and even greater values than those previously reported by us for PI fibers deposited onto Kevlar [10,13]. The differences observed can be attributed to the arrangement of functional groups during the electrospinning process, which modifies the membranes’ structure and consequently influences their moisture transport properties. In addition to the notably higher WVTR values, these new electrospun membranes are also very lightweight, flexible, and easy to handle and exhibit excellent mechanical properties. The results further indicate that there is a minimal difference in the WVTR among the samples PEEK/PI-5 and PEEK/PI-6, suggesting that the moisture level of the new membranes is not significantly influenced by the PI structure used.

According to the proposed purpose, the best results are achieved for the electrospun membranes PEEK/PI-1, PEEK/PI-3, andPEEK-PI-4. The testing is designed to simulate a real situation which mimics the scenario under certain clothing better, where moisture acts as an insulating layer. As a result, lower breathability values are typically observed because the air trapped between the fabric and the liquid water holds onto much of the vapor [42,43]. Thus, the side-chain phosphorus in DOPO introduces some flexibility, leading to a balance between porosity, the WVTR, and air permeability. On the other hand, according to the literature, highly breathable microporous membranes with a controlled pore size and high water repellency have been fabricated using fluorine-containing compounds [40,44,45]. Another of the microporous membranes, namely PEEK/PI-5, shows an extremely low air permeability (0.0768 m^3^/m^2^·min) and lower porosity (78.3%) despite it having a relatively high WVTR (2245.93 g/m^2^·day). This suggests that the main-chain phosphorus could create a denser fiber network that still allows moisture transfer due to the chemical interactions between the polymer and water vapor.

**Figure 4:**
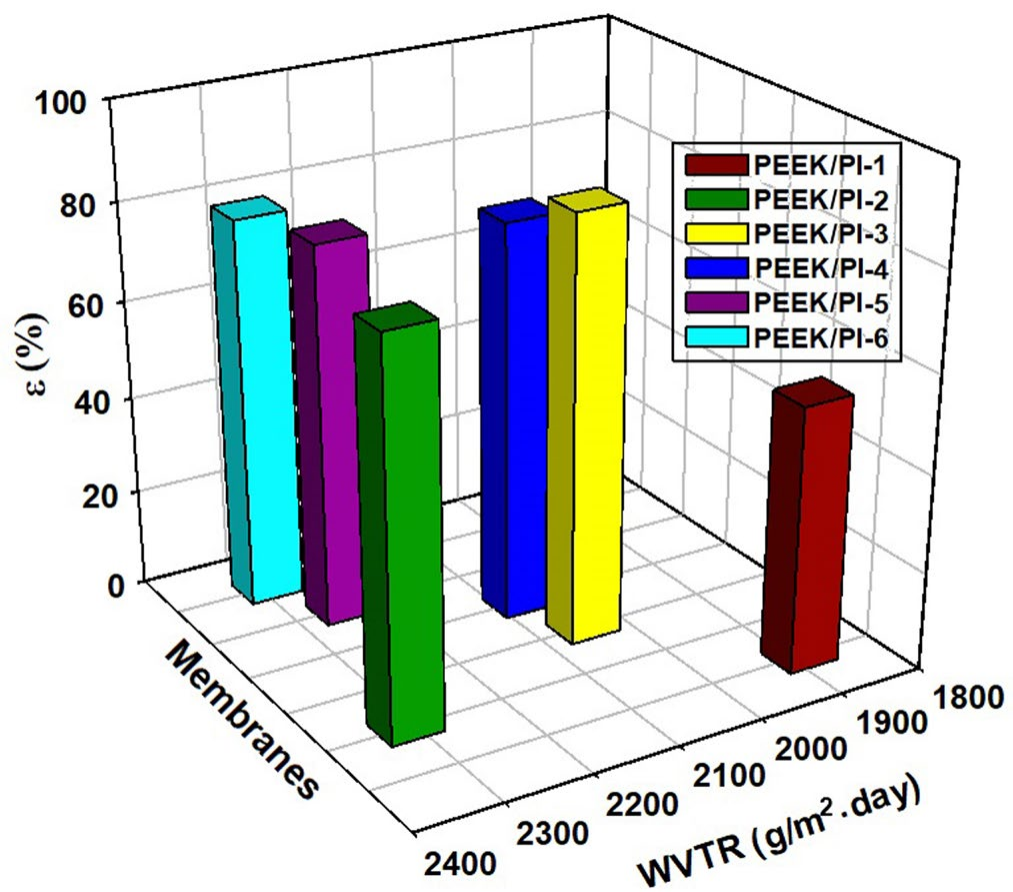
Proportionality between the transfer of water vapor and the porosity of the electrospun PEEK/PI (1–6) membranes.

Generally, a higher porosity allows for a greater WVTR because more pores can facilitate the transport of vapors. However, other factors like the volume fraction of voids or pores in the membrane, distribution, and membrane material can also play a role. In fact, an increase in the WVTR was observed with increasing inter-spaces resulting from the correlation between the average fiber diameter and the void volume fraction in the membrane (Figure 4). Thus, the plot also shows that higher-porosity membranes generally correspond to higher WVTR values, as expected due to the increased void space allowing for more vapor transfer. However, given that there are some variations between samples with a similar porosity, we can state that other factors can influence the transfer rate of water vapor beyond the porosity. An exception occurs in the case of PEEK/PI-1, which stands out from the other samples in having the lowest porosity and WVTR.

One of the main concerns for materials used in protective items like sportswear, medical gear, and military uniforms is their ability to protect the skin from harmful substances, toxic agents, and many other things [46,47,48]. While these materials provide a protective barrier, they can also prevent perspiration, leading to discomfort. In this sense, a lot of research has focused on developing membranes that act as barriers to liquids and harmful agents while maintaining adequate breathability [41,49,50]. However, hydrophilic materials, which tend to swell in the presence of water, must be carefully managed to avoid significant changes in the membrane structure under high humidity, as excessive swelling could compromise the protective barrier and allow toxic chemicals to pass through. Therefore, breathable porous membranes that block liquids are often favored to ensure adequate body perspiration. However, one key challenge is preventing liquids from seeping into the porous structure. Achieving this requires a careful balance between the pore size and the material’s level of water resistance to keep liquids from entering the membrane.

A viable tool for evaluating the so-called membrane waterproofness consists of establishing the degree of wetting, which depends on the ability of liquid to spread onto the surface, expressed as a function of the surface free energy, *ΔG_w_* (Equation (4)). As is known, the water contact angle, *θ*_water_, is an indicator of the wettability of a film surface [51,52]. Measuring the contact angle at the interface between the membrane surface and water provides a direct and quantitative estimate of the spreading, and the approach of membrane surface energy permits a correlation of the total surface tension of water (*γ_lv_* = 72.80 mN/m) with its chemical nature.

(4) $$\;\Delta G_{w}=-\gamma_{lv}(1+\cos\theta_{water})$$

**Figure 5:**
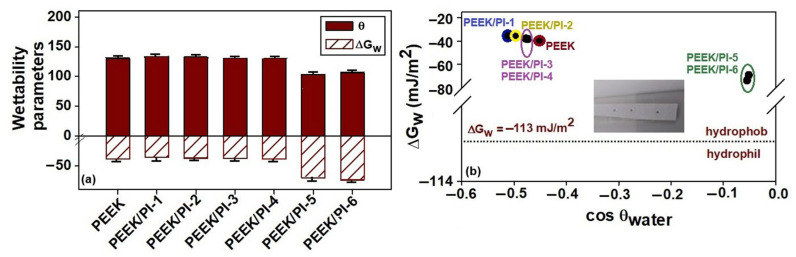
Wettability parameters: (**a**) water contact angle (*θ_water_*, degrees) and surface free energy (*ΔG_w_*, mJ/m^2^) of the electrospun PEEK/PI (1–6) membranes and (**b**) the hydrophilic/hydrophobic balance at the interface of the membrane surface–water. The insert picture indicates the degree of wettability of one of the membranes studied after 5 min through recording the contact angle.

Figure 5a displays a graphical representation of the contact angle and surface free energy values for the electrospun membranes based on PEEK/PIs (1–6). According to the literature, the surface of a polymer can be considered more hydrophilic with *ΔG_w_* < −113 mJ/m^2^, whereas when *ΔG_w_* > −113 mJ/m^2^, it should be considered more hydrophobic (Figure 5b). Considering these statements, the nature of the polymers and the modifications applied to the studied systems (PEEK/PIs (1–6)) determine a slight increase in the contact angles and a corresponding decrease in their surface wettability compared to the PEEK sample. Thus, all samples exhibit a hydrophobic nature, with contact angles slightly higher or comparable to that of pure PEEK. An exception is noted for the PEEK/PI-5 and PEEK/PI-6 samples, which have lower contact angle values (103.27° and 107.11°), which means they are less hydrophobic compared to the other samples (Figure 5). This could be due to the specific polyimide structure in PI-5 and PI-6, which have functional groups that enhance water attraction, leading to lower hydrophobicity.

Also, for example, in Figure 5b, the inserted image shows the recording of the contact angle after 5 min of measurement. As can be seen, after this time, water droplets remain on the surface of the material for an extended period and roll off the surface of the electrospun membrane when it is tilted at an angle of 7°. Therefore, given that chemistry and morphology are two key issues, their effects on the waterproofness of the membranes are examined. The wetting characteristics of the material surfaces studied can be altered by the orientation of the polymer chains and the rearrangement of functional groups on or within the membrane surfaces following the electrospinning process. This modification is driven by an increase in the local polar moments. We can conclude that the variation in the wettability parameters (the contact angle and surface free energy), which influence both comfort and safety, indicates that blending PEEK with different polyimides (PI-1 to PI-6) can effectively tune the hydrophilic/hydrophobic balance within the resulting material to provide superior barrier properties and enhanced resistance to moisture penetration and material–skin interaction, properties useful for targeted applications.

The interplay between enhanced surface hydrophobicity and a controlled structure directly influences a membrane’s permeability profile, achieving an optimal balance between moisture management and selective air flow, making these membranes suitable for applications in protective clothing, where an optimal combination of breathability and moisture control ensures wearer comfort, while enhanced barrier properties provide protection against external environmental factors. Based on the previously assessed properties of the water vapor transmission rate (WVTR) and wettability, the incorporation of PEEK into electrospun polyimide membranes offers a tailored approach to optimizing their permeability.

In traditional protective clothes, breathable materials are thus combined to offer a physical barrier for protection, but often, these clothing solutions involve higher permeability, generating temporary protection. In these situations, it is necessary that the multifunctional materials obtained have limited permeability. A practical way to eliminate this major risk is to evaluate this parameter by measuring the time required to see whether water permeates the membrane both under no applied pressure and with an applied pressure of 20 kPa or higher (Table 5). In certain instances, the water has been recirculated to assess whether the penetration time remained constant.

It was observed that under no applied pressure, water penetrated the PEEK/PI-1 membrane after 360 s and the PEEK/PI-6 membrane after 880 s. Besides PEEK/PI-1 and PEEK/PI-6, the other membranes could be classified as Class 6 of protective clothes according to EN 14,325 because the breakthrough time was higher than 480 min [53].

**Figure 6:**
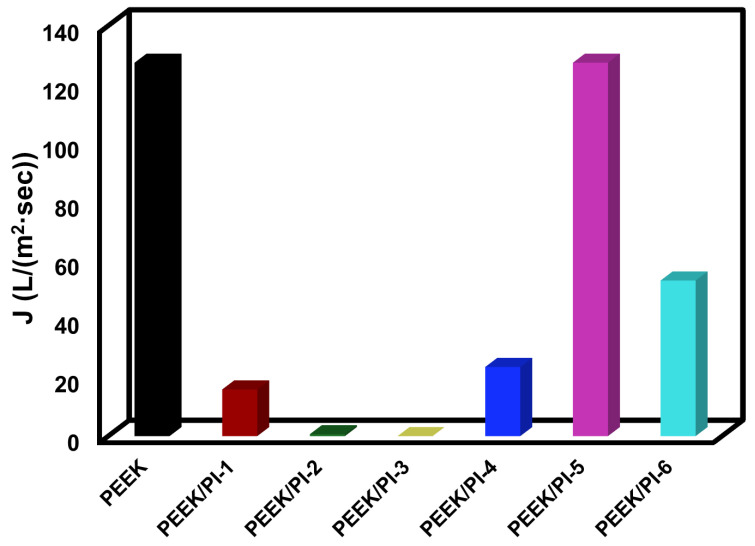
Permeation rate of the electrospun PEEK/PI (1–6) membranes.

When pressure was not applied to these membranes, breakthrough did not occur. When a pressure of 20 kPa was applied, water permeated the membranes in the following order: PEEK/PI-5 after 5 s, PEEK after 11 s, PEEK/PI-6 after 20 s, PEEK/PI-4 after 44 s, PEEK/PI-1 after 53 s, and PEEK/PI-2 after 1260 s. Notably, the PEEK/PI-3 membrane did not allow water penetration even when the pressure was increased to 60 kPa; only at a pressure of 70 kPa was water penetration observed after 770 s. By recirculating the water and subsequently retesting the membranes, it was observed that the permeation time decreased with the second cycle. However, these decreases stabilized after the third recirculation cycle, indicating that while the initial application of pressure resulted in an expansion of some of the pores, this deformation did not progress further during the subsequent recirculation processes. The degree of permeability is largely influenced by the water concentration gradient across the membrane, which can vary based on changes in partial pressure [54]. The permeation rates of the membranes studied, calculated after the third recirculation cycle of water and when applying a pressure of 20 kPa, are presented in Figure 6.

In the context of materials designed for protective clothing, it is imperative that the breakthrough time be maximized while the penetration rate be minimized. This ensures optimal protection against water infiltration. The observations revealed that the PEEK/PI-2 and PEEK/PI-3 membranes exhibited waterproof properties, making them suitable candidates for incorporation into protective clothing, ideal for sports clothes, healthcare, firefighting, and defense. Their performance indicates a high resistance to water penetration, which is crucial for ensuring safety and effectiveness in hazardous environments.

### 3.4. Efficiency of Air Permeability and Fire Resistance of the Electrospun Membranes

As disclosed in the literature, many non-breathable materials have been used as chemical barriers for protective garments, e.g., protective boots, gloves, or respirators, including CPF3™ by Kappler and certain polymers (polyvinyl chloride (PVC), polyvinyl fluoride (PVDF), butyl rubber, nitriles, and elastomers) [45,50,55,56,57,58]. However, the effectiveness of air-impermeable chemical protection has fallen short of meeting wearers’ comfort requirements, which are normally determined by regulated perspiration. For this purpose, the air permeability, AP, is a parameter that indicates how easily air can pass through the material, but its optimal value is context-specific, balancing the need for breathability with the functional requirements of the final product. For example, fabrics designed to ensure sufficient breathability without compromising their protective features may have lower air permeability values, below 10 m^3^/m^2^·min, to provide better protection against the elements. Thus, among the membranes studied, PEEK/PI-3 (1.2586 m^3^/m^2^·min) has the highest air permeability, followed by PEEK/PI-2 (1.1171 m^3^/m^2^·min), and PEEK/PI-1 (0.7951 m^3^/m^2^·min) has the lowest among the polymers without phosphorus in their main chain. Higher values suggest more porous or breathable materials. Therefore, the high permeability observed in PEEK/PI-3 can be attributed to its high porosity (87.2%), resulting from the presence of the bulky -CF_3_ groups in the dianhydride. These groups disrupt the fiber structure, creating larger air gaps and thereby enhancing permeability. PEEK/PI-1 has the lowest porosity and therefore lower permeability. The side-chain phosphorus in DOPO likely leads to more tightly packed fibers, reducing the air flow. Interestingly, PEEK/PI-5 and PEEK/PI-6, which contain phosphorus in their main chain, show very low air permeability, especially PEEK/PI-5 (0.0768 m^3^/m^2^·min). The presence of phosphorus in the main chain increases their rigidity, making the polymer chains less flexible during electrospinning. This rigidity can lead to denser fibers or fibers with reduced interconnected porosity, which significantly decreases air permeability (Table 4).

While their impressive air permeability underscores their suitability for filtration and breathable materials, it is equally noteworthy that these PEEK/PI (1–6) membranes, despite their thinness, exhibit exceptional flame resistance. In fact, their ability to pass the UL-94 test and achieve a V-0 (non-flammable) classification is a significant achievement, particularly given their molecular structure and electrospun fiber properties. The UL-94 V-0 classification is the highest flammability rating, meaning that the material stops burning within 10 s after ignition and does not drip flaming particles. This level of fire resistance can be largely attributed to the inclusion of phosphorus, which plays a crucial role in enhancing the flame retardancy of these materials. The presence of phosphorus, both in the side chains (as seen in PEEK/PI-1–PI-4 with DOPO) and in the main chains (as in PEEK/PI-5 and PEEK/PI-6), is a well-known flame-retardant mechanism [12,15]. Phosphorus-containing compounds promote char formation during combustion, which acts as a protective barrier, slowing down the spread of flames and reducing heat release [59,60,61]. In side-chain phosphorus structures, like those in PEEK/PI-1–PI-4, the phosphorus likely increases the char yield while maintaining some degree of flexibility and processability in the polymer. Main-chain phosphorus, as seen in PEEK/PI-5 and PEEK/PI-6, enhances the rigidity and thermal stability of the polymers, providing even greater resistance to thermal degradation and flame propagation. Moreover, the combination of phosphorus with the polyimide backbone, which itself is known for its thermal stability and inherent flame resistance, further contributes to the exceptional fire-retardant properties of these membranes [15,62]. Polyimides have high glass transition temperatures and can withstand significant thermal stress without breaking down. When phosphorus is integrated into this already robust structure, it creates a synergistic effect that makes the material highly resistant to ignition and flame spread. Another factor contributing to the membranes’ non-flammability, despite their thinness, is the high porosity and fibrous structure of the electrospun membranes. While high porosity might initially seem to increase the surface area available for combustion, the phosphorus-induced char formation and thermal stability actually help to prevent the material from burning. The thin fiber morphology also promotes the rapid dissipation of heat, preventing localized areas from reaching temperatures high enough to sustain combustion. Thus, even though these PEEK/PI membranes are thin, the combination of phosphorus chemistry, the thermal stability of the polyimides, and the unique porous fiber structure enables them to perform exceptionally well in the UL-94 test, earning the V-0 classification. This result highlights the effectiveness of the molecular design in achieving superior flame retardancy, even in materials with minimal thickness. The combination of advanced molecular design, phosphorus chemistry, and the inherent thermal stability of the polyimides has enabled these PEEK/PI membranes to achieve exceptional flame-retardant properties, demonstrating that even ultra-thin, porous materials can meet the highest safety standards without compromising performance.

## 4. Conclusions

This study demonstrates that electrospun membranes made from PEEK and phosphorus-containing polyimides (PIs (1–6)) offer a balance between protective and breathable qualities suitable for high-performance protective textiles. In this context, this research highlights the importance of precise structural and morphological adjustments in the electrospun membranes to achieve a balance between breathability and impermeability. Such properties are crucial in preventing overheating and moisture buildup, enhancing wearer comfort, maintaining physiological well-being, and providing protection in extreme conditions. The quantified results of this study can be summarized as follows:

- The electrospun PEEK/PI (1–6) membranes exhibit high water vapor transmission rate (WVTR) values, ranging from 1889.59 ± 110.73 to 2311.20 ± 206.58 g/m^2^·day, which exceed those reported for firefighter clothing materials and PI fibers on Kevlar. These high WVTR values indicate the materials’ ability to breathe, thus enhancing comfort.
- The correlation between the WVTR and the porosity of the electrospun membranes suggests that changes in their membrane structure during the electrospinning process enhance their suitability for protective clothing applications by optimizing their breathability.
- Measuring the contact angle at the interface between the membrane surface and water provided a direct, quantitative assessment of spreading behavior, which reflected the moisture dynamics typically encountered under garments and indicated the materials’ waterproof properties. The prolonged presence of water droplets on the membranes’ surface confirms their superior barrier properties and enhanced resistance to moisture penetration, an essential property for protective clothing in harsh environments, such as firefighter clothing.
- Furthermore, testing of water penetration demonstrated that the PEEK/PI-2 and PEEK/PI-3 membranes exhibited the best waterproof properties, making them suitable candidates for incorporation into protective clothing designed for firefighting.
- The air permeability values ranged from 0.0768 to 1.2586 m^3^/m^2^·min, with lower values observed in the membranes with finer pores, indicating reduced porosity and smaller inter-fiber spaces.
- The combination of phosphorus chemistry, the thermal stability of the polyimides, and the unique porous fiber structure enabled the PEEK/PIs membranes to perform exceptionally well in the UL-94 test, achieving the V-0 classification. This result demonstrates that incorporating phosphorus into the polymer backbone, as seen in PEEK/PI-5 and PEEK/PI-6, enhances the rigidity and thermal stability, providing greater resistance to thermal degradation and flame propagation.

Overall, the ability to optimize transport properties such as the WVTR, wettability, and water and air permeability, combined with enhanced flame resistance, positions the PEEK/PI electrospun membranes as promising candidates for advanced protective clothing, particularly for applications in demanding environments where breathability, moisture control, and fire resistance are essential.

## Figures and Tables

**Table 1:** Thermal properties and flame resistance of the phosphorus-containing polymers PIs (1–6).

Polymer	T_g_ ^a^ (°C)	T_5%_ ^b^ (°C)	T_10%_ ^c^ (°C)	η_750 °C_ ^d^ (%)	LOI
PI-1	-	304	437	49.70	37.4
PI-2	-	394	433	57.20	40.4
PI-3	-	200	272	42.50	34.5
PI-4	-	179	272	50.70	37.8
PI-5	191.91	460	485	47.29	36.4
PI-6	227.27	447	485	61.36	42.0

^a^ The glass transition temperature, determined from the DSC curves; ^b^ the temperature at which the polymer loses 5% of its weight; ^c^ the temperature at which the polymer loses 10% of its weight; ^d^ carbonaceous residue measured at 750 °C.

**Table 2:** Electrospinning conditions, viscosity, and average fiber diameters of the electrospun PIs (1–6).

Codes	Optimal Electrospinning Conditions	Viscosity (Pa·s)	Average Fiber Diameters (nm)
PI-1	25%, 12 cm ^a^, 50 µL/min ^b^, 19.2 kV ^c^, 36% ^d^, 23 °C ^e^	0.190	288 ± 68
PI-2	28%, 14 cm ^a^, 15 µL/min ^b^, 20 kV ^c^, 25% ^d^, 23 °C ^e^	0.560	512 ± 18
PI-3	30%, 14 cm ^a^, 15 µL/min ^b^, 22.5 kV ^c^, 25% ^d^, 23 °C ^e^	0.023	“beads”
PI-4	30%, 14 cm ^a^, 15 µL/min ^b^, 22.5 kV ^c^, 25% ^d^, 23 °C ^e^	0.029	“beads”
PI-5	25%, 10 cm ^a^, 15 µL/min ^b^, 20 kV ^c^, 25% ^d^, 23 °C ^e^	0.420	322 ± 66
PI-6	18%, 15 cm ^a^, 15 µL/min ^b^, 22.5 kV ^c^, 35% ^d^, 25 °C ^e^	0.230	298 ± 44

^a^ The distance between the tip of the needle and the collector; ^b^ the flow rate of the solution; ^c^ voltage; ^d^ relative humidity; ^e^ temperature.

**Table 3:** Electrospinning conditions along with the average fiber diameters for PEEK/PI (1–6) membranes.

Codes	Optimal Electrospinning Conditions	Average Fiber Diameters (μm)
PEEK	15% PEEK, 20 cm ^a^, 20 μL/min ^b^, 22 kV ^c^, 49% ^d^, 25 °C ^e^	0.211 ± 0.048
PEEK/PI-1	15% (PEEK)/0.1 g (PI-1), 16 cm ^a^, 20 μL/min ^b^, 24.6 kV ^c^, 37% ^d^, 24 °C ^e^	0.157 ± 0.032
PEEK/PI-2	15% (PEEK)/0.1 g (PI-2), 20 cm ^a^, 20 μL/min ^b^, 22 kV ^c^, 39% ^d^, 25 °C ^e^	0.234 ± 0.047
PEEK/PI-3	15% (PEEK)/0.1 g (PI-3), 20 cm ^a^, 20 μL/min ^b^, 22.8 kV ^c^, 40% ^d^, 24 °C ^e^	0.135 ± 0.029
PEEK/PI-4	15% (PEEK)/0.1 g (PI-4), 20 cm ^a^, 20 μL/min ^b^, 21.8 kV ^c^, 40% ^d^, 23 °C ^e^	0.300 ± 0.028
PEEK/PI-5	15% (PEEK)/0.1 g (PI-5), 20 cm ^a^, 20 μL/min ^b^, 21.7 kV ^c^, 40% ^d^, 25 °C ^e^	0.293 ± 0.069
PEEK/PI-6	15% (PEEK)/0.1 g (PI-6), 20 cm ^a^, 20 μL/min ^b^, 22 kV ^c^, 40% ^d^, 25 °C ^e^	0.252 ± 0.062

^a^ The distance between the tip of the needle and the collector; ^b^ the flow rate of the solution; ^c^ voltage; ^d^ relative humidity; ^e^ temperature.

**Table 4:** Transport parameter values evaluated for electrospun membranes based on phosphorus polymers: porosity (*ε*), water vapor transport through the material (WVTR), and air permeability (AP).

Membranes	Porosity (%)	WVTR (g/m^2^·day)	Air Permeability·10^5^ (m^3^/m^2^·min)
PEEK	60.3	2011.58 ± 256.30	0.7900 ± 0.85
PEEK/PI-1	54.7	1889.59 ± 110.73	0.7951 ± 1.97
PEEK/PI-2	79.9	2311.20 ± 206.58	1.1171 ± 1.55
PEEK/PI-3	87.2	2047.50 ± 142.15	1.2586 ± 1.42
PEEK/PI-4	81.4	2074.99 ± 153.66	1.0590 ± 1.05
PEEK/PI-5	78.3	2245.93 ± 318.29	0.0768 ± 0.82
PEEK/PI-6	79.8	2292.47 ± 264.90	0.1448 ± 1.25
Kevlar *		816.30 ± 21.20	24.716 ± 0.65
Kevlar/PI *		797.20 ± 23.50	14.245 ± 0.78

* Data from the literature [10].

**Table 5:** Water permeation time through the studied membranes under the conditions of no pressure being applied (initially, i) or a pressure of 20 kPa being applied.

Membranes	Number of Water Recirculation	Applied Pressure (kPa)	Water Permeation Time (s)
PEEK	i	0	-
	1	20	11
	2	20	8
	3	20	5
PEEK/PI-1	i	0	360
	1	20	53
	2	20	48
	3	20	40
PEEK/PI-2	i	0	-
	1	20	1260
	2	20	960
	3	20	960
PEEK/PI-3	i	0	-
	1	20	-
	1	40	-
	1	60	-
	1	70	770
PEEK/PI-4	i	0	-
	1	20	44
	2	20	27
	3	20	27
PEEK/PI-5	i	0	-
	1	20	5
PEEK/PI-6	i	0	880
	1	20	20
	2	20	14
	3	20	12

## Data Availability

Data are contained within the article.
